# Severe Disseminated Cryptococcosis Leading to Multi-organ Failure in a Renal Transplant Patient: A Case Report

**DOI:** 10.2174/0115734056353324250302180953

**Published:** 2025-03-25

**Authors:** Daniela Sanchez-Lobo, Paulina Espinosa-Zerecero, Ricardo Cebrian-Garcia, Marcos Garcia-Nava, Fernando Cano-Garcia, Maria-del-Carmen Garcia-Blanco, Ernesto Roldan-Valadez

**Affiliations:** 1 Universidad Anahuac Mexico Sur, 01840, Mexico City, Mexico; 2 Hospital Angeles Acoxpa, Education Department, 14308, CDMX, Mexico; 3 Hospital Angeles Acoxpa, Radiology Department, 14308, CDMX, Mexico; 4 Hospital Angeles Acoxpa, Intensive Care Unit, 14308, CDMX, Mexico; 5 Hospital Angeles Acoxpa, Nephrology Unit, 14308, CDMX, Mexico; 6 Hospital Angeles Acoxpa, Department of Pathology, 14308, CDMX, Mexico; 7 Division of Research, Instituto Nacional de Rehabilitacion 'Luis Guillermo Ibarra Ibarra', 14389, Mexico City, Mexico; 8 Department of Radiology, I.M. Sechenov First Moscow State Medical University (Sechenov University), 119992, Moscow, Russia

**Keywords:** Cryptococcosis, Renal transplant, Opportunistic infections, Immunosuppression, Multi-organ failure, Fungal infections, Post-transplant complications, *Cryptococcus gattii*, Pneumonectomy, Case report

## Abstract

**Background:**

Cryptococcosis is a severe but rare opportunistic fungal infection predominantly affecting immunocompromised individuals, such as post-transplant patients. The diagnosis is frequently delayed due to non-specific symptoms and lower incidence than other fungal infections.

**Case Report:**

A case of a 50-year-old male renal transplant recipient who developed disseminated cryptococcosis complicated by multi-organ failure is presented. Despite adherence to international treatment guidelines, the patient's condition rapidly deteriorated due to the extensive immunosuppression required for transplant rejection management. The patient developed pneumonia and was diagnosed with disseminated cryptococcosis on the 10th day of hospitalization, with *Cryptococcus gattii* identified in the pulmonary system and pleura. The patient underwent multiple interventions, including bronchoscopy, lobectomy, and pneumonectomy. Despite aggressive treatment, the infection progressed, leading to severe complications, such as neurological decline, gastrointestinal bleeding, and ultimately, multi-organ failure. The patient passed away after 53 days of hospitalization.

**Conclusion:**

This report highlights the importance of early diagnosis and multidisciplinary management in post-transplant patients with suspected opportunistic infections. The high mortality associated with disseminated cryptococcosis, particularly in severely immunosuppressed patients, underscores the need for vigilance and prompt intervention to improve patient outcomes.

## 1 INTRODUCTION

Pneumonia is a prevalent complication among post-transplant patients. Despite this, the diagnosis and management of fungal pneumonia are often delayed, as these infections are more frequently attributed to viral and bacterial pathogens [1]. This delay can result in the postponement of specific antifungal therapies, leading to severe complications. Studies indicate that 50-70% of patients exhibit disseminated or extrapulmonary infections at diagnosis [2].

Cryptococcosis, a significant opportunistic fungal infection, is the third most common fungal infection in post-transplant patients, following aspergillosis and candidiasis. It accounts for approximately 8-10% of all fungal infections in this population [3, 4]. The incidence is notably higher among renal and heart transplant recipients [5], with rates ranging from 0.4% to 5.8% in renal transplant patients, as reported in recent studies, likely due to the substantial use of monoclonal antibodies and corticosteroids in these procedures [6].

Cryptococcosis significantly contributes to morbidity and mortality, with a mortality rate of 14% for disseminated cases, escalating to 70% for infections caused by *Cryptococcus gattii*. Age plays a critical role, as older individuals are reported to have a higher risk of severe outcomes due to age-related immunosuppression and comorbidities [5, 7]. Although expected in immunosuppressed patients with solid organ transplants, the occurrence of cryptococcosis alongside renal failure and pneumonectomy due to massive atelectasis is unusual. It is rare to encounter such a complex interplay of pathological processes triggered by a fungal infection with a relatively low global incidence.

This case report presents a unique opportunity to examine a patient experiencing these multifaceted conditions. We aim to share insights into the diagnosis, treatment, clinical progression, and outcome of a 50-year-old male patient with disseminated cryptococcosis. In addition to medical imaging, such as CT scans and pathology reports, comprehensive assessments, including bronchoscopy, bronchoalveolar lavage, pleural fluid antigen testing, and laboratory investigations, were conducted during the patient's hospital stay, highlighting the multifaceted diagnostic approach required for cryptococcosis in immunocompromised individuals.

This case study is of significant academic interest to clinicians and postgraduate medical students. It provides a detailed review of disseminated cryptococcosis and explores recent advancements in its diagnosis and treatment. The insights from this case can enhance the understanding of complex post-transplant infections and underscore the importance of a multidisciplinary approach in managing such challenging cases. All diagnostic and therapeutic procedures were conducted following approval by the institution's ethics committee and after obtaining informed consent from the patient. This study adhered to the ethical principles of the Declaration of Helsinki and relevant national guidelines.

## 2 CASE REPORT

A 50-year-old male from Mexico City with a notable medical history presented to the hospital. His family history was significant for prostate cancer and diabetes mellitus, both reported by his father. His past medical interventions comprised an appendectomy during childhood, a renal transplant in 2013, and the creation of a brachiocephalic arteriovenous fistula in 2017. The patient was a smoker for 10 years, smoking 20 cigarettes daily, resulting in a smoking index of 10. Additionally, he had chronic degenerative conditions, including chronic kidney disease (CKD), classified as KDIGO stage G5, secondary to glomerulonephritis of unknown origin. He had received a renal transplant from a living-related donor in 2017, developed type 2 diabetes mellitus secondary to the renal transplant, was treated with glargine insulin, and had systemic arterial hypertension managed with telmisartan and amlodipine.

In November 2023, the patient was hospitalized due to acute kidney injury, classified as KDIGO stage 3, and mixed rejection of the renal transplant, identified as acute cellular rejection (Banff 2019 IA) and antibody-mediated rejection. His treatment included rituximab, intravenous immunoglobulin, and methylprednisolone. After a 30-day hospital stay, he was discharged with outpatient management involving tacrolimus, mycophenolic acid, and prednisone. Due to impaired renal function, he commenced weekly hemodialysis sessions.

Approximately two weeks before his emergency department visit in 2024, the patient began experiencing symptoms of asthenia, adynamia, general malaise, intermittent expectorant cough, dyspnea, and non-cyanotic episodes. A chest X-ray performed by his pulmonologist indicated consolidation in the left lower lobe, suggestive of pneumonia. Despite outpatient treatment with moxifloxacin, his symptoms worsened, leading to his presentation to the emergency department 22 days after the onset of symptoms.

Upon arrival, his vital signs were recorded, which included a blood pressure of 133/70 mmHg, heart rate of 85 bpm, respiratory rate of 30 rpm, oxygen saturation of 95%, and temperature of 36.6°C. The physical examination revealed a conscious, cooperative male showing signs of pain, mild pallor of the mucous membranes and skin, and a symmetrical thorax with normal heart sounds. However, decreased air entry was noted at the left lung base, accompanied by Velcro crackles. His extremities were intact, with symmetrical coloration and normal temperature, though ++ edema was observed in the lower limbs.

Laboratory investigations revealed several abnormalities, including hemoglobin 9.07 g/dL, hematocrit 28.3%, leukocytes 6.59 K/uL, neutrophils 93.4%, glucose 231.5 mg/dL, blood urea nitrogen 58 mg/dL, urea 124.1 mg/dL, creatinine 5.22 mg/dL, AST 94.1 U/L, ALT 557.3 U/L, direct bilirubin 0.58 mg/dL, alkaline phosphatase 216 U/L, GGT 212 U/L, lactate dehydrogenase 446.2 U/L, sodium 130.7 mEq/L, chloride 98.8 mEq/L, magnesium 1.5 mEq/L, C-reactive protein 101.9 mg/L, cystatin C 4.46 mg/L, and a filtration rate of 17 mL/min.

**Fig. (1):**
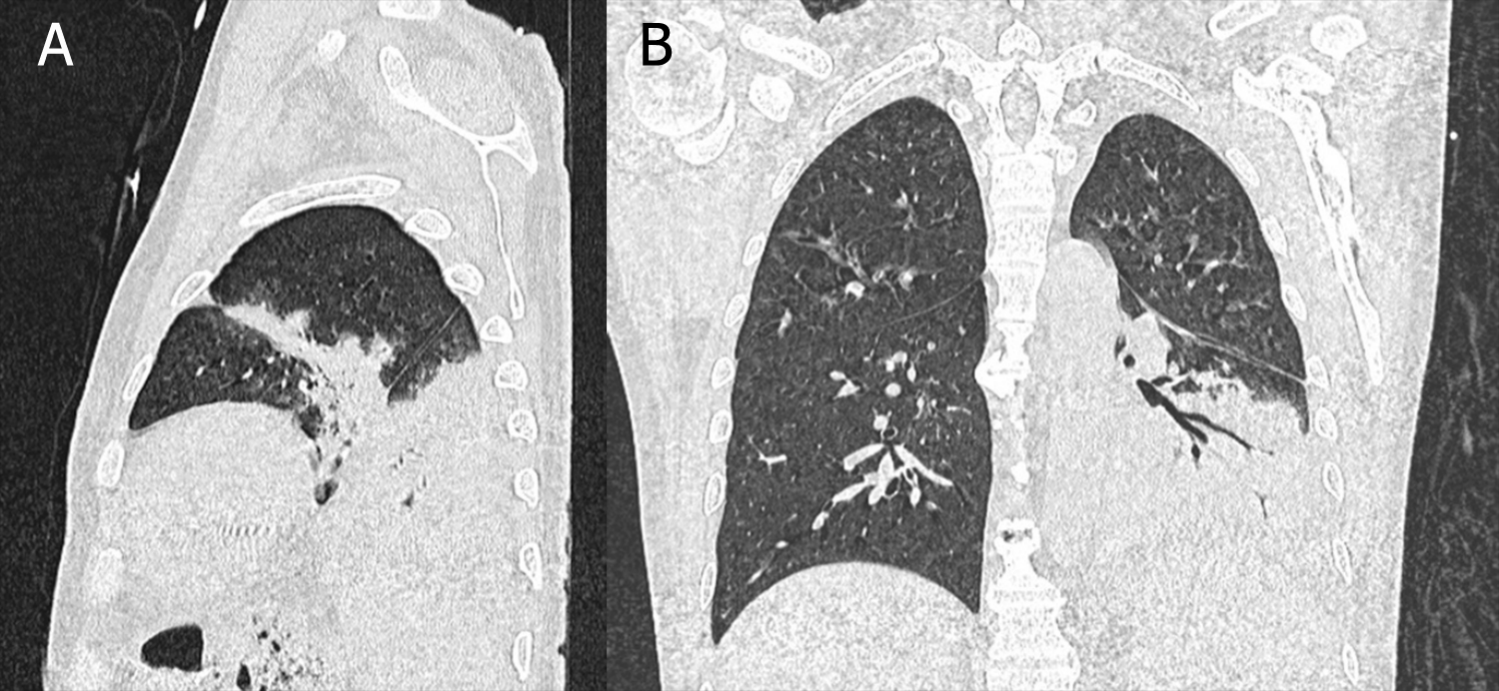
Non-contrast chest CT. Sagittal (**A**) and coronal (**B**) sections demonstrate an enlarged cardiac silhouette, inflammatory axillary and mediastinal lymphadenopathy, and areas of pulmonary consolidation in both lung fields. The most significant consolidation is located in the posterior segment of the left lower lobe, accompanied by an air bronchogram. Bilateral pleural effusion can also be observed, consistent with an active infectious process.

The patient was diagnosed with anemic and uremic syndromes, necessitating immediate dialysis. He was admitted to the intermediate care unit, where urgent hemodialysis sessions began. Given his respiratory symptoms, a pulmonology consultation was requested. A non-contrast chest CT scan identified multiple pneumonia foci, bilateral pleural effusion, cardiomegaly, axillary and mediastinal adenopathies, and bilateral renal atrophy (Fig. **1**). A pneumonia molecular panel from sputum tested positive for *Staphylococcus aureus*, indicating community-acquired pneumonia. Consequently, the infectious diseases team initiated antibiotic therapy with linezolid and moxifloxacin.

**Fig. (2):**
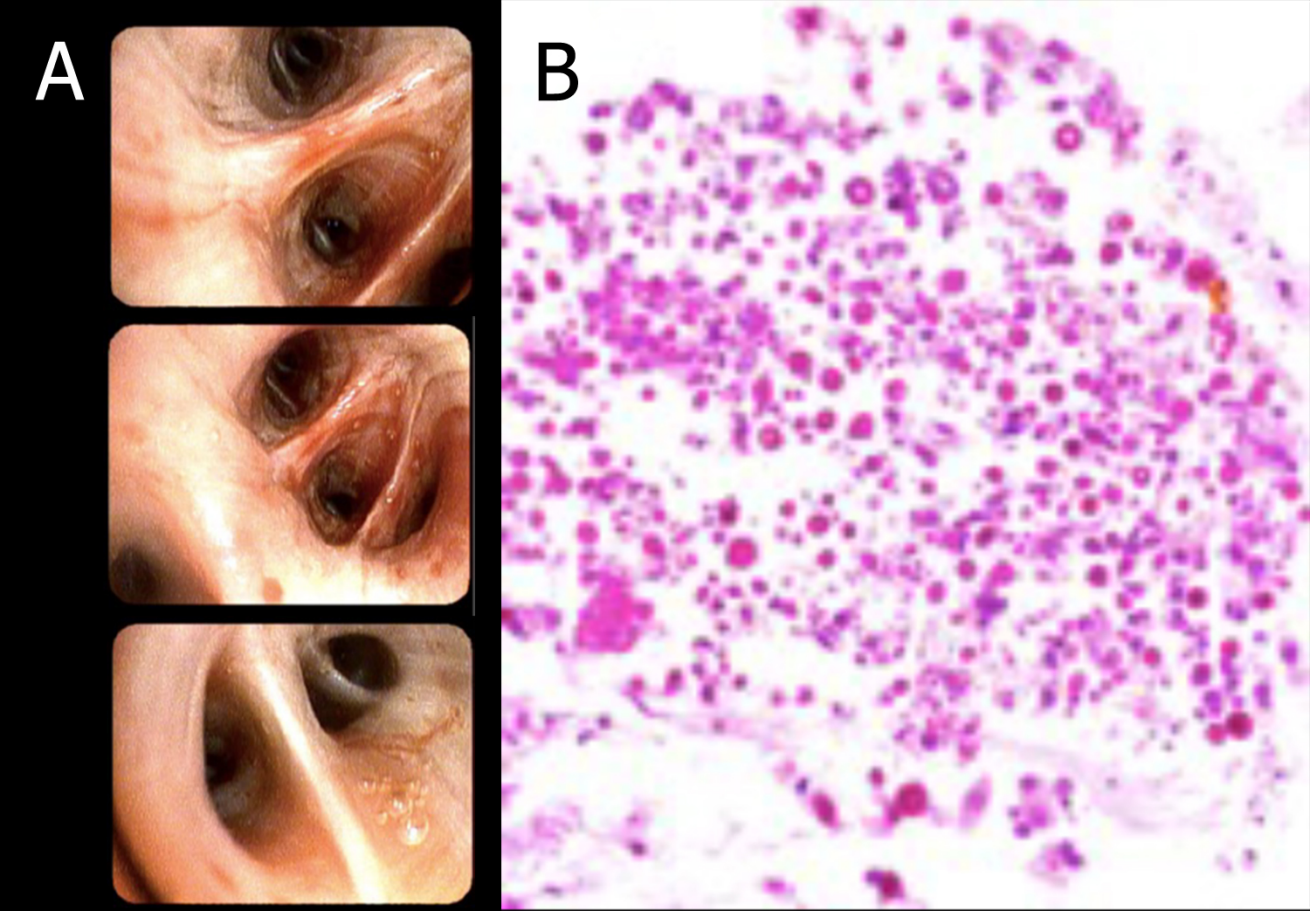
Diagnostic bronchoscopy and histopathological section. (**A**): Diagnostic bronchoscopy showing abundant purulent secretions originating from the left lower lobe. The bronchial mucosa is erythematous. (**B**): Transbronchial biopsy showing bronchial mucosa with moderate acute and chronic inflammation associated with the presence of fungal microorganisms morphologically consistent with cryptococcosis.

Considering the imaging findings and the patient's risk factors, a diagnostic bronchoscopy was conducted two days post-admission (Fig. **2A**). Abundant purulent secretions were observed in the left lower lobe, prompting the collection of bronchoalveolar lavage and five left transbronchial biopsies (Fig. **2B**). Pathology confirmed the presence of *Cryptococcus gattii* on the second day after hospitalization, leading to discontinuation of moxifloxacin. The initiation of therapeutic management included liposomal amphotericin B at a dose of 5 mg/kg/day and fluconazole at 800 mg/day. Linezolid at 600 mg every 12 hours was also continued for the *Staphylococcus aureus* pneumonia.

**Fig. (3):**
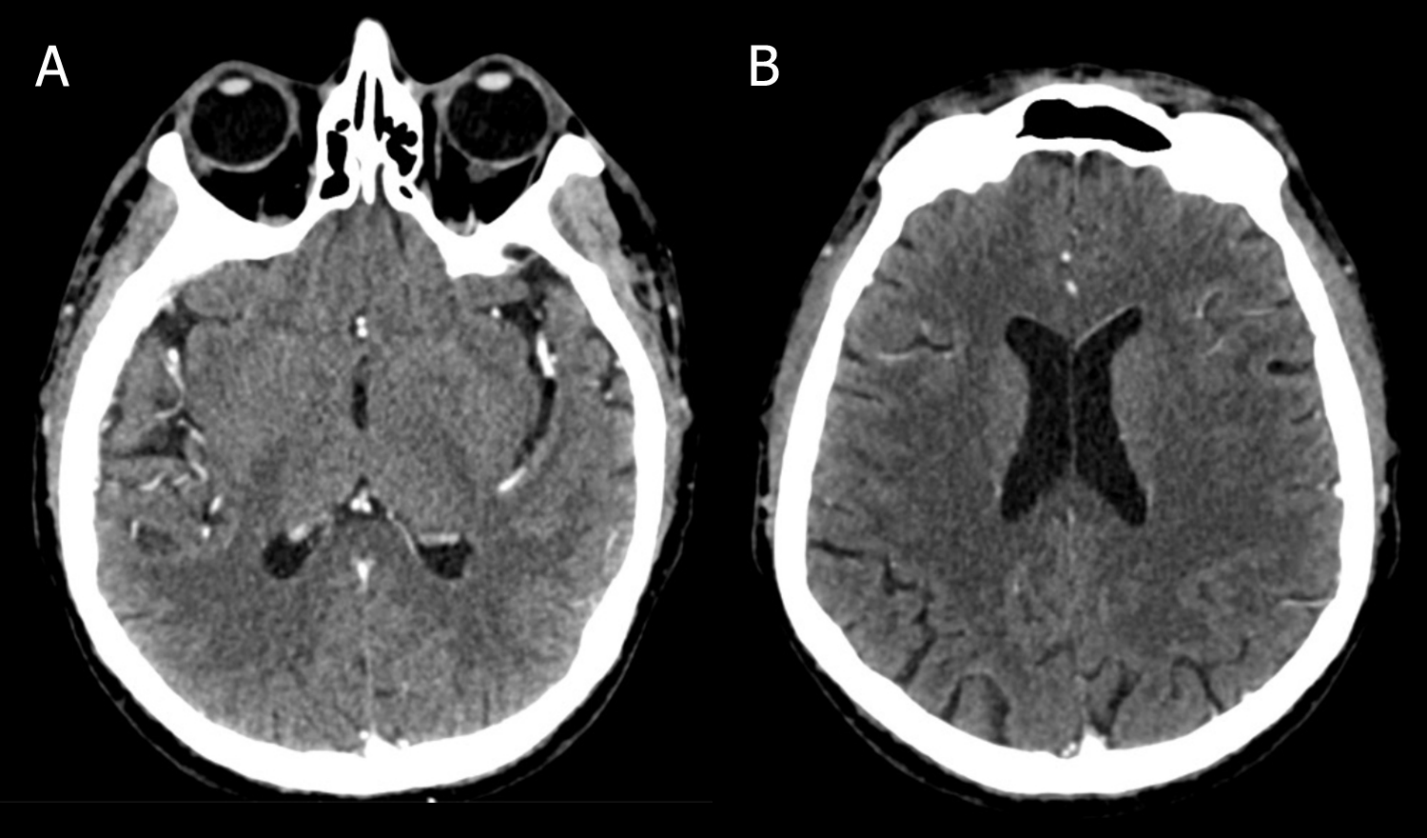
Non-contrast cranial CT. Axial images show no evidence of acute intracranial pathology. (**A**) Axial image at the level of the M2 segment of the middle cerebral artery. (**B**) Axial image at the level of the lateral ventricles. Gray-white matter differentiation is preserved, with well-maintained sulci, fissures, and brain cisterns. The ventricular system appears broad but within physiological limits. Physiological calcifications can be noted in the choroid plexus of the occipital horns. The overall impression was angiosclerosis without additional significant findings.

Due to chronic renal failure, the patient underwent hemodialysis every other day. The diagnosis of disseminated cryptococcosis prompted a thorough investigation to detect the presence of the microorganism in other systems. HIV serology returned non-reactive, with a CD4 count of 151 lymphocytes/μL. Serological tests for hepatitis B and C were also negative. Given *Cryptococcus gattii's* potential for central nervous system (CNS) invasion, a non-contrast cranial CT scan and lumbar puncture were performed, which showed no evidence of CNS involvement (Fig. **3**). A contrast-enhanced chest CT showed no new abnormalities.

Ten days into his hospitalization, routine blood tests revealed a significant drop in platelet count to 13,000/μL. A bone marrow aspirate and biopsy were performed to determine if this decline resulted from an inflammatory response or fungal infiltration of the bone marrow. Results indicated that the platelet reduction was due to a massive systemic inflammatory response, as no *Cryptococcus gattii* was detected in the bone marrow culture.

Despite eight days of targeted antibiotic therapy, the patient developed fever spikes exceeding 38°C on the tenth day of hospitalization. Both central and peripheral blood cultures confirmed disseminated cryptococcosis, with Gram stains testing positive for yeast.

**Fig. (4):**
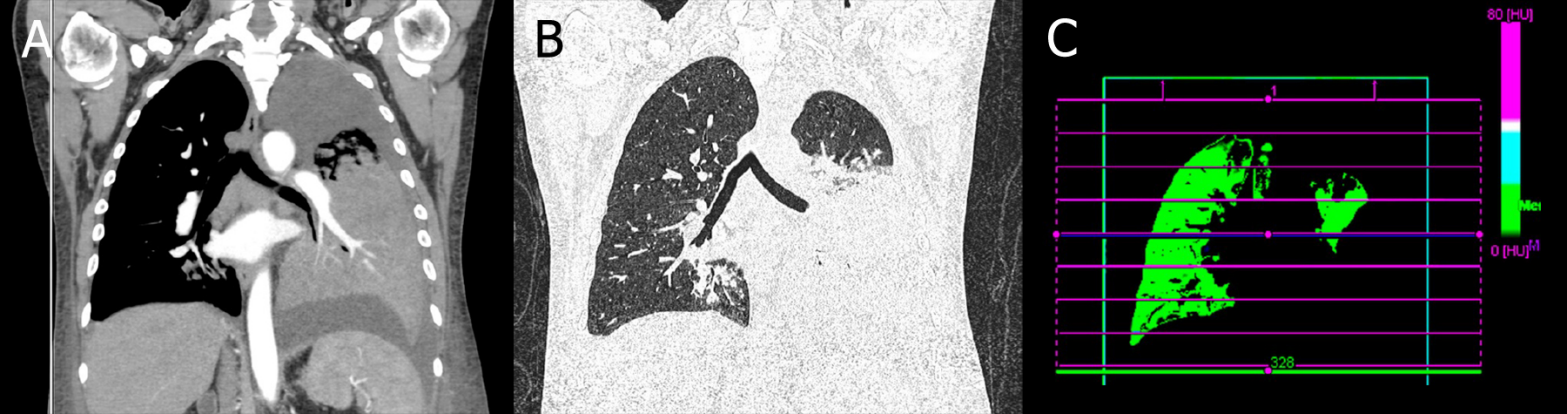
Simple and contrast-enhanced thoracic CT. (**A** and **B**): Mediastinum and lung windows showing small nodular images in the paratracheal regions on both sides of the mediastinum. The right lung parenchyma displays consolidations in the lateral segment (S4), superior segment (S6), and anterior basal segment (S8). The left lung shows infiltration and consolidation in the apicoposterior segment (S1+2) extending to the base. Additionally, there is a right pleural effusion with passive atelectasis and a loculated left pleural effusion. Compared to the previous CT scan, there was an enlarged cardiac silhouette, particularly of the left ventricle, and the newly observed changes in lung segments and pleural effusion. (**C**): A more than 80% decrease in left lung perfusion can be seen. Ventilation-perfusion scans show coronal cuts with ventilation areas represented in green, and the left lung lobectomy is visible in the lingula area.

Following a contrast-enhanced thoracic CT scan, which revealed perfusion abnormalities in the left lung (Fig. **4A**-**4C**), a decision was made to perform a left lower lobectomy due to unresponsive pneumonia. A multidisciplinary team optimized the patient's preoperative condition, including nephrology, cardiology, hematology, pulmonology, and infectious disease specialists. The antibiotic regimen was adjusted to include liposomal amphotericin B, flucytosine, and meropenem, and blood transfusions were administered. The patient was also diagnosed with heart failure with an ejection fraction of 32%.

**Fig. (5):**
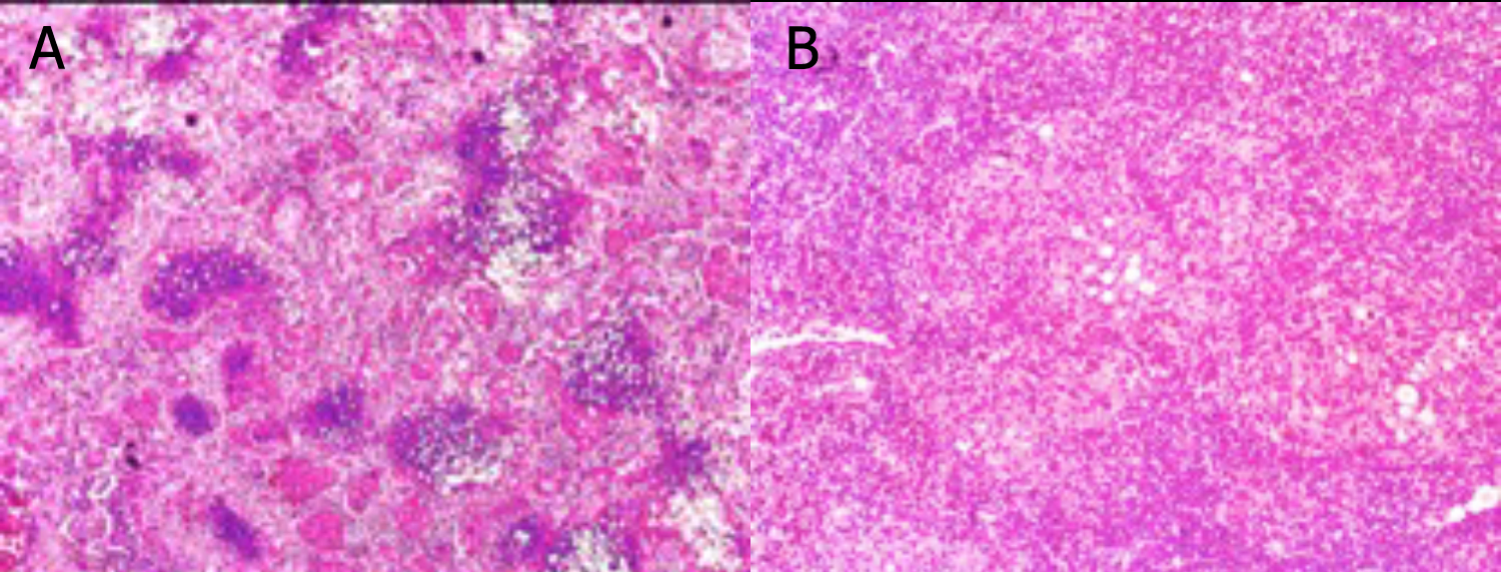
Histological section. (**A**): Pulmonary parenchyma showing necrotizing granulomatous inflammation associated with cryptococcosis affecting 100% of the pulmonary lobe. (**B**): Histological section of pulmonary lymph node showing necrotizing granulomatous lymphadenitis associated with cryptococcosis.

Forty-three days after the onset of symptoms, a left lower lobectomy was performed via thoracotomy, revealing a bleeding pulmonary hilum with loss of anatomical structures due to multiple enlarged lymph nodes and an infectious mass lesion in the lingula. Pathology confirmed necrotizing granulomatous inflammation associated with cryptococcosis, affecting the entire pulmonary lobe (Fig. **5A**) and necrotizing granulomatous lymphadenitis (Fig. **5B**). Left pleural fluid tested positive for *Cryptococcus gattii* antigen. Postoperatively, the patient was admitted to the intensive care unit with diagnoses of postoperative left lower lobectomy, resolving hypovolemic shock, CKD stage 5 on hemodialysis, living-donor kidney transplant recipient, mixed rejection, post-transplant diabetes mellitus, immunocompromised pneumonia caused by *S. aureus*, and pulmonary and disseminated cryptococcosis.

**Fig. (6):**
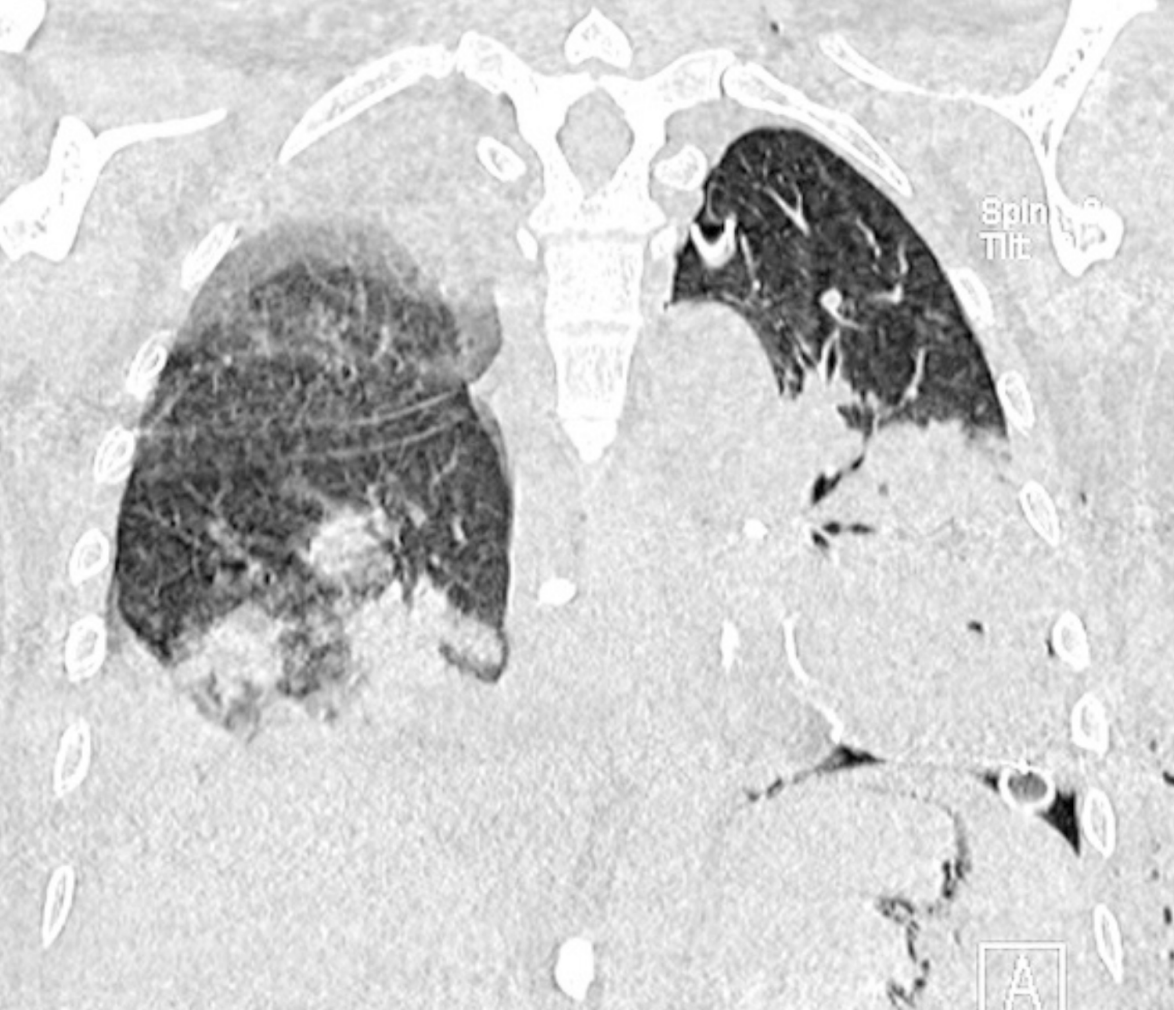
Thoracic CT scan showing bilateral basal alveolar opacities, consolidation areas, and bilateral pleural effusion with right-sided predominance.

In the first two days post-lobectomy, the patient remained hemodynamically stable and tolerated antifungal treatment well, with no significant inflammatory response (CRP 49 mg/L, procalcitonin 2 ng/mL). However, on the third day, CRP levels rose to 152 mg/L, and the patient's respiratory function deteriorated. A thoracic CT scan showed bilateral basal alveolar opacities, consolidation, and bilateral pleural effusion, predominantly on the right side (Fig. **6**). A thoracocentesis was performed, draining 200 mL of right pleural effusion, which tested positive for *Cryptococcus gattii*.

On the 23rd day of hospitalization, the patient experienced a neurological decline, with mixed delirium, increased productive cough, respiratory distress, tachycardia (106 bpm), and tachypnea (35 rpm). Due to combined neurological and respiratory deterioration, orotracheal intubation was performed. Subsequent ultrasound revealed a complicated right pleural effusion with suspected pleural infiltration.

One day after intubation, the patient experienced an upper gastrointestinal bleed of approximately 1000 cc. Endoscopy identified a giant gastric ulcer in the fundus, covered with fibrin. A thoracoscopy was performed to drain the right pleural effusion, resulting in significant clinical improvement, and the patient was extubated two days later.

**Fig. (7):**
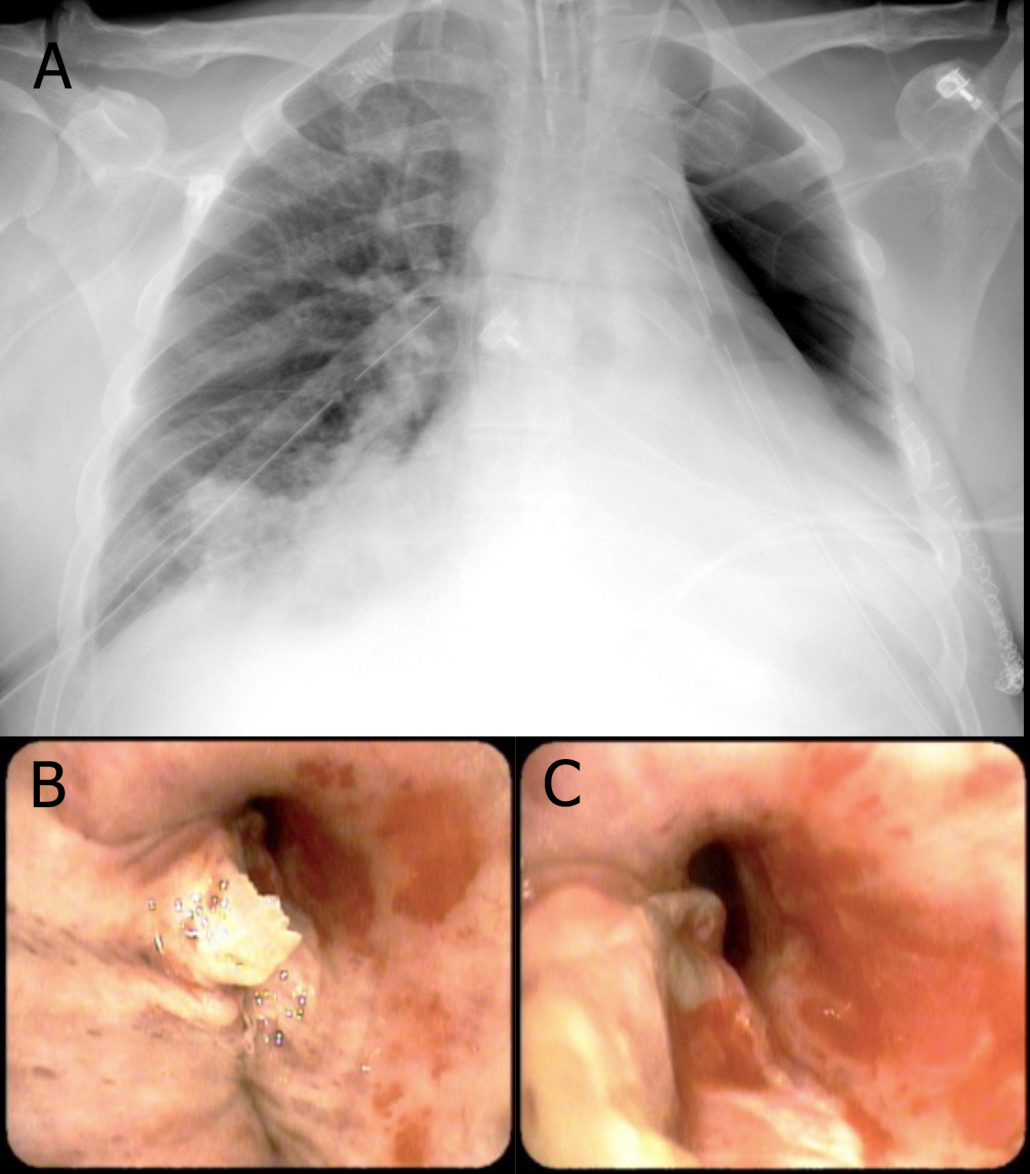
(**A**) Portable chest X-ray showing left thoracotomy surgical changes, pleural tube placement with metallic sutures, and pulmonary consolidation in the lower right and middle and lower left lung. Left pleural effusion obscures the costophrenic angle. Prominent pulmonary hila with loss of vascular morphology could be observed. (**B** and **C**): Second diagnostic bronchoscopy showing abundant old hematic secretions in the right bronchial tree. Cotton III stenosis of the left upper lobe segmental bronchus suggestive of pulmonary mycosis infiltration.

However, 24 hours post-extubation, the patient again experienced respiratory deterioration, accompanied by massive left-sided atelectasis (Fig. **7A**), necessitating a second orotracheal intubation and a second diagnostic bronchoscopy (Fig. **7B** and **C**) with bronchoalveolar lavage and biopsy. Severe stenosis of the left upper lobe bronchial lumen, suggestive of pulmonary mycosis infiltration, was observed.

**Fig. (8):**
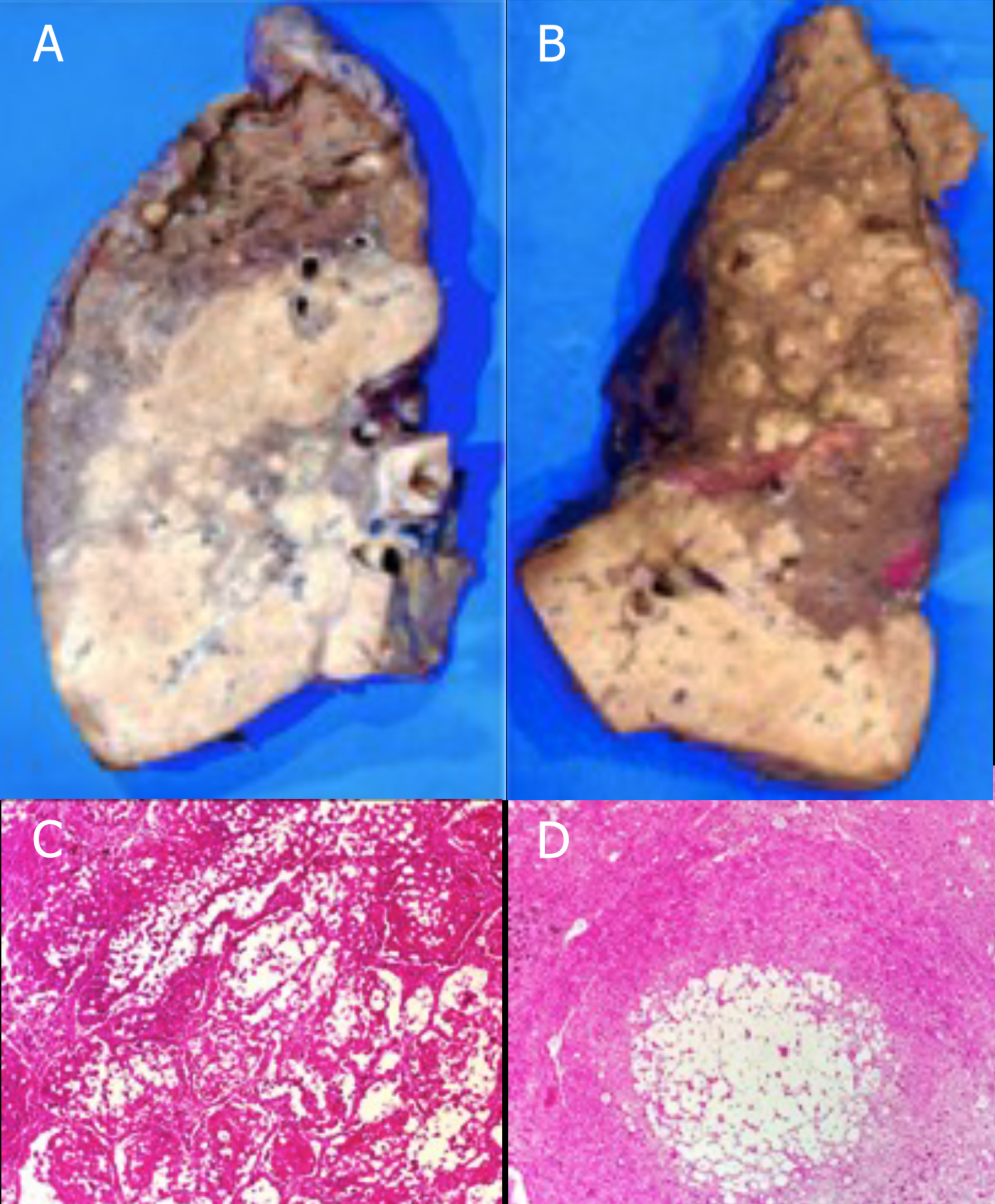
(**A** to **C**): Macroscopic and microscopic sections of left lung showing pulmonary parenchyma with acute and chronic abscessed granulomatous inflammation associated with cryptococcosis in the process of degeneration affecting 70% of the pulmonary lobe. (**D**): Histological section of hilar lymph node showing necrotizing granulomatous lymphadenitis associated with cryptococcosis in the process of degeneration.

Due to severe stenosis, a left pneumonectomy was performed on the 38th day of hospitalization. Histopathological examination revealed pulmonary parenchyma with acute and chronic abscessed granulomatous inflammation associated with cryptococcosis, affecting 70% of the pulmonary lobe (Fig. **8A-C**) and necrotizing granulomatous lymphadenitis (Fig. **8D**). Bronchial secretion cultures tested positive for *Enterococcus faecium*, indicating a nosocomial superinfection.

On the 39^th^ day, the patient was diagnosed with gastrointestinal dysfunction due to poor tolerance of parenteral nutrition. Hemoglobin, hematocrit, and platelet levels continued to decrease without apparent bleeding. A colonoscopy on the 42nd day revealed lower gastrointestinal bleeding secondary to diverticulitis, ulcerative proctitis with active bleeding, and internal hemorrhoids.

**Fig. (9):**
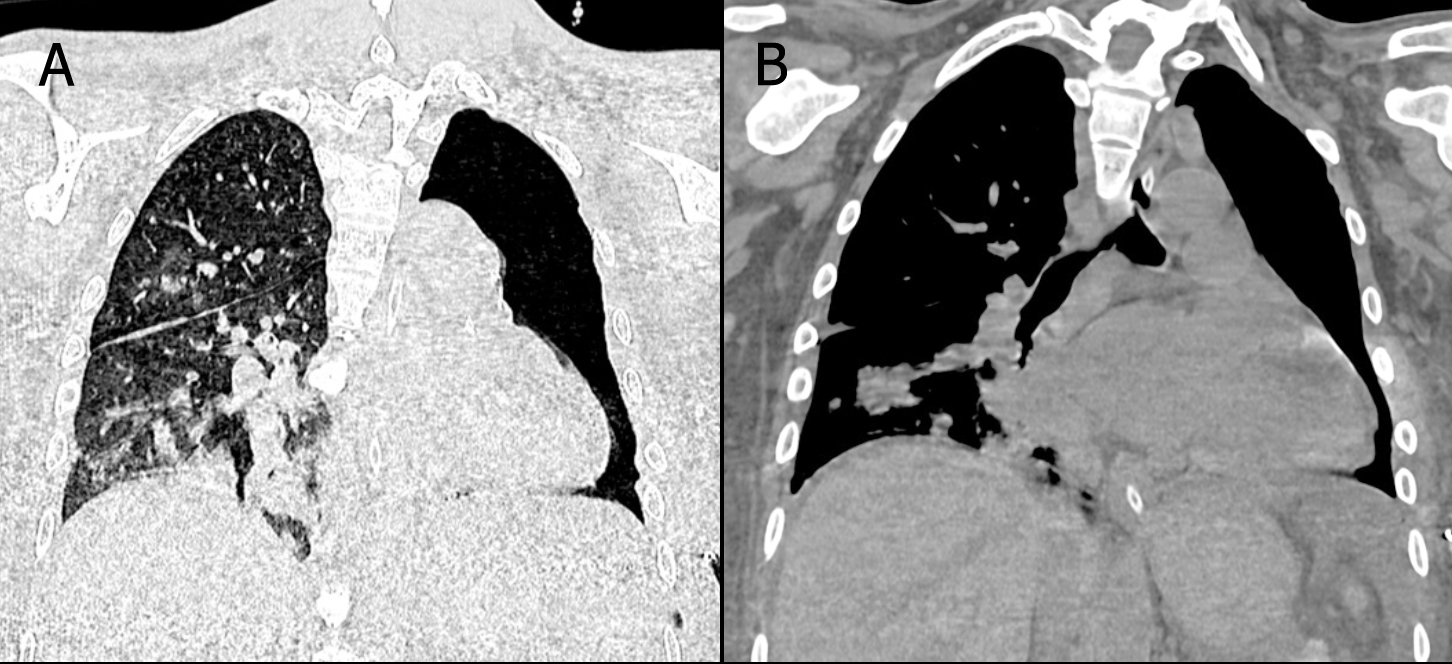
(**A** and **B**): Non-contrast thoracic CT showing post-surgical changes from left pneumonectomy with pneumothorax and minimal residual consolidated lower lobe lung tissue with surrounding fluid. Decreased volume of right pulmonary consolidation areas and associated pleural effusion with interfissural fluid. Focal rounded pneumonitis areas with ground-glass appearance predominantly in the right upper lobe.

On the 44^th^ day, a percutaneous tracheostomy was performed via bronchoscopy due to prolonged orotracheal ventilation (13 days) and ongoing diaphragmatic dysfunction. Multiple purulent secretions were found in the right bronchial tree, and a follow-up thoracic CT scan revealed new areas of pneumonitis in the right lung, suggestive of a superimposed infectious process (Fig. **9A****, ****9B**). Five days post-procedure, *Trichosporon asahii* was identified as the causative agent of the new infection from a bronchial secretion sample.

**Fig. (10):**
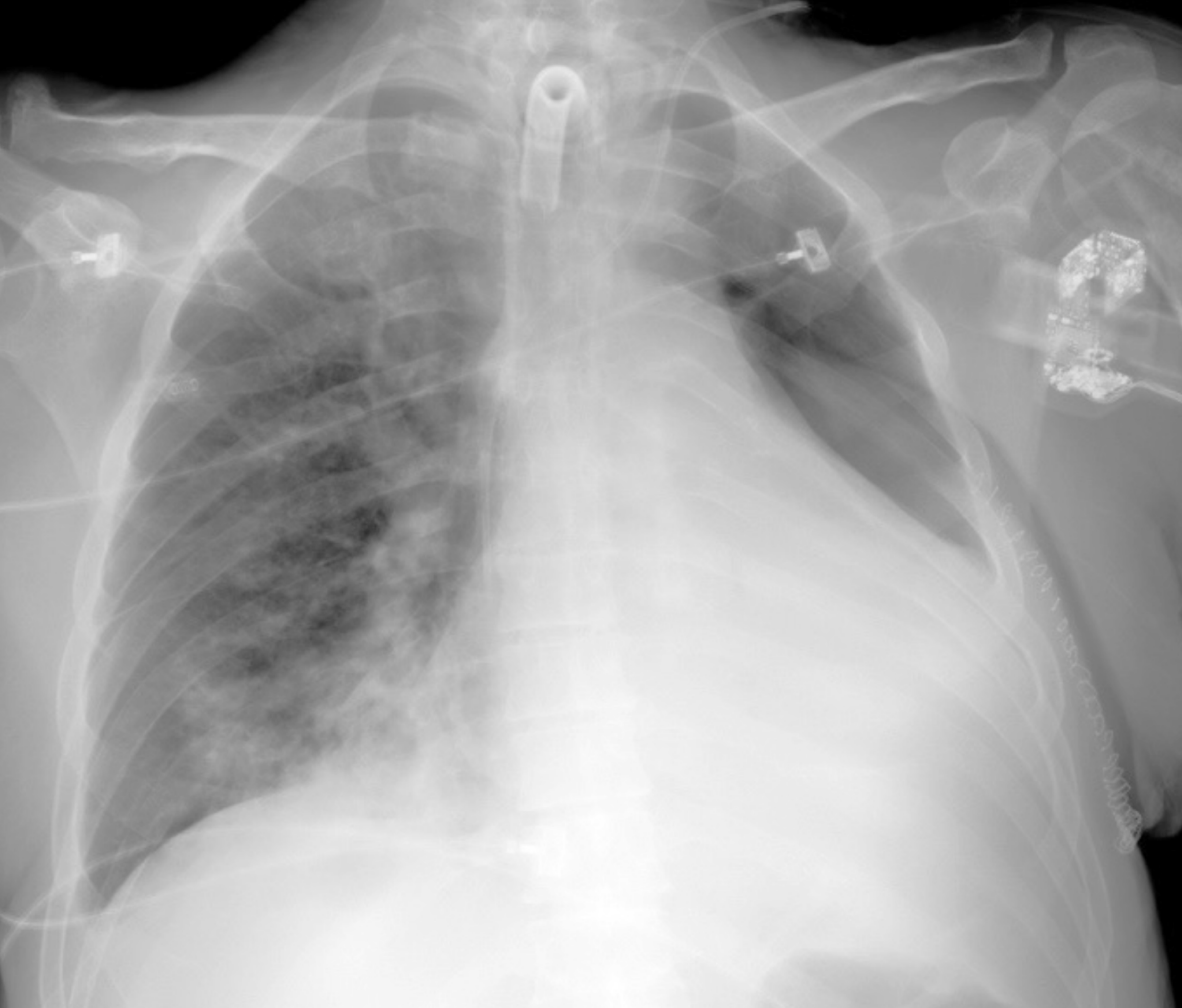
AP chest X-ray showing a collapsed left lung with air density in the pulmonary cavity, obliteration of the cardiophrenic recess, and retraction of mediastinal structures to the left.

The patient's neurological status further deteriorated, leading to a vegetative state declared by the neurology team on the 50th day of hospitalization, based on transcranial Doppler ultrasound, cranial CT scan, and electroencephalogram findings. Despite multidisciplinary efforts, the patient's condition worsened with multiple organ failure and no improvement in imaging studies (Fig. **10**). The family signed an advance directive, and palliative care was initiated. The patient passed away on the 53rd day of hospitalization, 75 days after symptom onset, surrounded by family.

## 3 DISCUSSION

### 3.1 Overview of Cryptococcus

*Cryptococcus*, a genus comprising approximately 30 species, includes the highly pathogenic *Cryptococcus neoformans* and *Cryptococcus gattii*, which pose significant risks to humans. These fungi are characterized by their oval or round yeast forms and reproduce primarily through budding. A critical factor contributing to their virulence is their complex polysaccharide capsule, which is both antiphagocytic and immunosuppressive. This capsule, containing elements, such as glucuronoxylomannan (GXM) and glucuronoxylomannan galactan, allows the fungi to effectively evade the host's immune response. Moreover, *Cryptococcus* species secrete extracellular enzymes, like phospholipases, ureases, and laccase, which further enhance their survival within the host [8, 9].

### 3.2 Epidemiology and Risk Factors

Globally, *Cryptococcus* infections account for significant morbidity and mortality, with an estimated 220,000 cases of cryptococcal meningitis annually among HIV-infected individuals alone and a high burden in solid organ transplant recipients, where incidence ranges from 0.2% to 5% depending on the geographic region [3, 8]. These infections predominantly affect young adults, males, and individuals with HIV [7]. Historically confined to tropical and subtropical regions, cryptococcal infections have expanded in recent years, appearing across North and South America, Asia, Australia, and Africa [9]. Acute mortality rates, especially in cases complicated by meningoencephalitis, can range between 35-40%, leading to hundreds of thousands of deaths annually [8]. Risk factors for cryptococcal disease include HIV infection, immunosuppressive therapies, solid organ transplants, neoplasms, lymphomas, autoimmune diseases, and hematological disorders [8].

### 3.3 Pathogenesis

*Cryptococcus neoformans* is typically found in bird droppings, whereas *Cryptococcus gattii* is associated with decaying plant matter, particularly eucalyptus trees. Infection usually occurs through inhaling dried yeast cells into the pulmonary alveoli, where alveolar macrophages attempt to phagocytize the yeast. In immunocompetent individuals, this response often resolves the infection. However, the infection can become latent in immunocompromised hosts, leading to pneumopathy and the potential for dissemination to other organs, most notably the central nervous system (CNS) [9, 10].

### 3.4 Clinical Manifestations

The clinical presentation of cryptococcosis varies depending on the infected organ, with cryptococcal meningitis being the most critical and life-threatening manifestation. Symptoms can range widely from general malaise, severe headache, and behavioral changes to fever, cough, weight loss, and pneumonia [10]. This broad spectrum of symptoms often complicates the diagnostic process, delaying timely and appropriate treatment.

*Cryptococcus* infections are frequently misdiagnosed due to their nonspecific symptoms and overlapping clinical and radiological features with other conditions, such as tuberculosis, lung cancer, and bacterial pneumonia. Thambidurai *et al*. [11] emphasized that cryptococcal pneumonia often mimics tuberculosis or malignancy, requiring biopsy for confirmation. Similarly, Salazar *et al*. [6] reported that 27.5% of cryptococcosis cases in hospitalized patients were misdiagnosed, leading to delayed treatment and an 8.8% increase in 90-day mortality among HIV-negative patients. Wang *et al*. [12] highlighted that limited nodules or masses with mild symptoms are common causes of misdiagnosis, resulting in unnecessary surgical interventions in immunocompetent patients and contributing to higher morbidity rates due to delayed diagnosis.

### 3.5 Humoral Rejection in Transplant Patients

Humoral rejection, also known as antibody-mediated rejection, predominantly occurs when alloantibodies target human leukocyte antigens (HLA) class I and class II present on the vascular endothelium of transplanted organs. Additional targets may include ABO antigens, angiotensin II type I receptor antibodies, and platelet-specific antigens. Sensitization to anti-HLA antibodies often results from prior exposures, such as pregnancies or blood transfusions [13]. HLA alloantibody-mediated rejection is a leading cause of allograft failure, especially in renal transplant patients, where it accounts for nearly 60% of late rejections occurring after the first year post-transplant. These rejections typically present as subacute dysfunction, which may or may not be accompanied by proteinuria and de novo donor-specific HLA antibodies [14, 15].

The absence of standardized clinical guidelines for effectively managing humoral rejection contributes to its poor prognosis. Patients with antibody-mediated rejection face a graft loss risk up to four times higher than those without it [13, 14]. Treatment options have been explored, including rituximab, intravenous immunoglobulins (IVIG), complement inhibitors, IL-6/IL-8 blockers, bortezomib, and splenectomy, but none have definitive scientific evidence supporting their efficacy. Thus, prevention remains the most effective strategy, focusing on avoiding HLA incompatibilities and adhering to treatment protocols [15].

In the case presented, rituximab and intravenous immunoglobulin were utilized. Rituximab, a monoclonal antibody, targets the CD20 antigen on B lymphocytes, inducing cell apoptosis [14, 16]. IVIG is an immunomodulator and immunosuppressant during the acute phase of antibody-mediated rejection [17]. The combined use of these therapies aims to suppress the recipient's immune response to rejection, thus reducing the likelihood of graft loss. However, this immunosuppressive approach can induce hypogamma-globulinemia, significantly increasing susceptibility to opportunistic infections and other complications due to a weakened immune system [18].

### 3.6 Clinical Integration

The patient's initial hospitalization in November 2023, due to acute kidney injury (KDIGO 3) and mixed rejection of the renal transplant, marks a critical point in his clinical course. Immunosuppressive medications were necessary to prevent organ rejection.

A case reported by L. Fernández Rodríguez *et al*. in 2005, published in Nefrología, described a 32-year-old woman with a deceased donor renal transplant undergoing immunosuppressive therapy with prednisone, cyclosporine, and mycophenolate. She developed disseminated cryptococcal neuroinfection, which was diagnosed on the seventh day of hospitalization through lumbar puncture and blood antigen tests. Treatment in the intensive care unit included liposomal amphotericin B and 5-flucytosine, later replaced with fluconazole. She was discharged after three weeks with significant clinical improvement [19].

The study concluded that cryptococcosis in renal transplant recipients is rare, with this case being the first documented over 27 years of kidney transplants. Cryptococcosis is more prevalent in heart transplant recipients due to the higher immunosuppressive levels required, with prevalence rates reported to range from 2% to 5%, depending on geographic and institutional factors [3]. The study highlighted a significant association between the use of steroids and other immunosuppressive drugs, such as tacrolimus, and the risk of cryptococcal infection. Patients with renal insufficiency have an impaired Th-1-mediated immune response against *Cryptococcus* species, resulting in greater dissemination to other organs and rapid disease progression [19].

In the present case, rituximab therapy likely contributed to prolonged B cell depletion, lasting at least 6 to 9 months in monotherapy patients and up to 18 to 24 months in those receiving continuous treatment with other drugs. Hypogammaglobulinemia, a known complication of rituximab therapy, significantly increases the risk of infection. Therefore, measuring baseline immunoglobulin levels before starting treatment is crucial, as well as monitoring these levels closely for at least one year post-treatment [17].

Upon the patient’s admission to the emergency department, laboratory results revealed immunosuppression associated with rituximab therapy, along with outpatient treatment involving tacrolimus (0.075 mg/kg/day) and mycophenolic acid (1 g twice daily). The clinical presentation of a longstanding infection, without a significant leukocyte count elevation and with high neutrophil and inflammation markers, supported this diagnosis. This state of immunosuppression likely facilitated the rapid disease progression and dissemination to the pleura and bloodstream. Despite the initiation of liposomal amphotericin B (5 mg/kg/day) and fluconazole (800 mg/day) after diagnosis, the disease progressed to multi-organ failure, ultimately leading to death.

### 3.7 Additional Clinical Considerations

Liver enzyme abnormalities observed on admission suggested chronic or hepatotoxic drug use, impacting liver function. After ruling out infectious causes, like hepatitis, the patient was diagnosed with drug-induced liver injury (DILI), a significant contributor to drug withdrawals from the market. Approximately 50% of acute liver failure patients develop DILI due to hepatotoxic drugs, and of these, 40% may require liver transplantation [20].

Protein-energy wasting (PEW), a reduction in protein and energy reserves, is another significant factor, encompassing malnutrition and catabolism [21]. PEW notably increases mortality in patients with renal disease by reducing lymphocyte counts and albumin levels [22].

A study in Madrid, Spain, used subjective global assessments and PEW criteria, including biochemical markers, muscle mass, BMI, and food intake, to demonstrate that proteinuria exceeding 10g in 24 hours and under 2.5g, combined with low total lymphocyte count, is a critical marker of poor nutrition, increased complications, and mortality in severely ill patients [21].

This case involved a rare and severe form of disseminated cryptococcosis, which resulted in multiple organ failure, including chronic kidney disease, extensive pulmonary involvement, and eventual multi-organ failure. Despite adherence to therapeutic guidelines and international standards, the complexity of the patient's condition necessitated continuous interventions, highlighting the limitations of current treatment protocols. The rapid deterioration observed was further exacerbated by underlying factors, such as malnutrition and protein-energy wasting, which significantly contributed to poor prognosis.

## CONCLUSION

This case report underscores the critical importance of early diagnosis and intervention in managing fungal infections, like cryptococcosis, especially in immunosuppressed patients, such as renal transplant recipients. The capacity of cryptococcosis to disseminate across multiple organ systems, coupled with its ability to mimic more common bacterial or viral infections, poses significant diagnostic challenges. Such complexities often result in delayed treatment, leading to severe complications that can be fatal.

This case highlights the necessity of a multidisciplinary approach in managing complex post-transplant infections. Early recognition of symptoms, a high index of suspicion for opportunistic infections, and prompt, targeted therapeutic interventions are vital to improving patient outcomes. Healthcare professionals must remain vigilant for signs indicating potential deterioration, especially in patients with a history of significant immunosuppression.

Ultimately, the tragic outcome of this case emphasizes the urgent need for ongoing research and education to advance the management of similar cases in the future. By sharing these insights, this report aims to provide valuable knowledge to clinicians and postgraduate medical students, fostering a more proactive and informed approach to caring for patients with complex post-transplant complications. Enhanced awareness and understanding of these infections can lead to earlier intervention, better management strategies, and improved patient survival.

## Declaration of Generative AI and AI-assisted Technologies in the Writing Process

While preparing this manuscript, the authors used Grammarly software to correct grammar and style. Following the use of this tool, all authors reviewed and edited the content to ensure accuracy and maintain responsibility for the final version of the publication. No other generative AI or AI-assisted technologies were used in the writing or preparation of this manuscript.

## Data Availability

The data and supportive information are available within the article.

## LIST OF ABBREVIATIONS

CKD: Chronic kidney disease
CNS: Central nervous system
CRP: C-reactive protein
CT: Computed tomography
DILI: Drug-induced liver injury
GXM: Glucuronoxylomannan
HLA: Human leukocyte antigen
IVIG: Intravenous immunoglobulin
KDIGO: Kidney Disease Improving Global Outcomes
PEW: Protein-energy wasting

## STANDARDS OF REPORTING

This manuscript followed the CARE guidelines, ensuring that all aspects of the case are reported comprehensively and transparently. These guidelines were adhered to when presenting the patient's clinical history, diagnostic procedures, interventions, outcomes, and follow-up.
